# Deciphering thyroid function and CIMT: a Mendelian randomization study of the U-shaped influence mediated by apolipoproteins

**DOI:** 10.3389/fendo.2024.1345267

**Published:** 2024-03-22

**Authors:** Ming-zhu Zhang, Cong Zhao, Xiao-ming Xing, Jie Lv

**Affiliations:** ^1^ Department of Nephrology and Endocrinology, Dongzhimen Hospital, Beijing University of Chinese Medicine, Beijing, China; ^2^ Department of Respiratory Disease, Dongzhimen Hospital, Beijing University of Chinese Medicine, Beijing, China

**Keywords:** thyroid hormones, Mendelian randomization, carotid intima-media thickness, apolipoprotein A-I, apolipoprotein B

## Abstract

**Background:**

Carotid Intima-Media Thickness (CIMT) is a key marker for atherosclerosis, with its modulation being crucial for cardiovascular disease (CVD) risk assessment. While thyroid function’s impact on cardiovascular health is recognized, the causal relationship and underlying mechanisms influencing CIMT remain to be elucidated.

**Methods:**

In this study, Mendelian Randomization (MR) was employed to assess the causal relationship between thyroid function and CIMT. Thyroid hormone data were sourced from the Thyroidomics Consortium, while lipid traits and CIMT measurements were obtained from the UK Biobank. The primary analysis method was a two-sample MR using multiplicative random effects inverse variance weighting (IVW-MRE). Additionally, the study explored the influence of thyroid hormones on lipid profiles and assessed their potential mediating role in the thyroid function-CIMT relationship through multivariate MR analysis.

**Results:**

The study revealed that lower levels of Free Thyroxine (FT4) within the normal range are significantly associated with increased CIMT. This association was not observed with free triiodothyronine (FT3), thyroid-stimulating hormone (TSH), or TPOAb. Additionally, mediation analysis suggested that apolipoprotein A-I and B are involved in the relationship between thyroid function and CIMT. The findings indicate a potential U-shaped curve relationship between FT4 levels and CIMT, with thyroid hormone supplementation in hypothyroid patients showing benefits in reducing CIMT.

**Conclusion:**

This research establishes a causal link between thyroid function and CIMT using MR methods, underscoring the importance of monitoring thyroid function for early cardiovascular risk assessment. The results advocate for the consideration of thyroid hormone supplementation in hypothyroid patients as a strategy to mitigate the risk of carotid atherosclerosis. These insights pave the way for more targeted approaches in managing patients with thyroid dysfunction to prevent cardiovascular complications.

## 1 Introduction

Atherosclerosis, the primary cause of global mortality, constitutes a chronic inflammatory disease affecting large and medium-sized arteries, leading to conditions such as ischemic heart disease, strokes, and peripheral vascular disease collectively termed cardiovascular disease (CVD) (1). The prevalence of total CVD cases nearly doubled from 271 million in 1990 to 523 million in 2019, resulting in 18.6 million CVD-related deaths in 2019, as reported by the latest Global Burden of Disease (GBD) study (2). Carotid intima-media thickness (CIMT) is a widely used surrogate marker for atherosclerosis, providing a simple, reproducible, sensitive, and noninvasive measure, and a robust predictor of future cardiovascular events (3). Identifying modifiable risk factors for CIMT is paramount.

Thyroid function is recognized for its multifaceted impact on the cardiovascular system (4). Elevated circulating thyroid hormone levels have been linked to hypertension and hypercoagulation, while decreased thyroid hormone levels may lead to hyperlipidemia and inflammation. Observational studies have consistently associated variations in thyroid function within the normal range with an increased risk of atherosclerosis (5), atrial fibrillation (6), stroke (7), heart failure, and mortality (8). The underlying mechanisms involve factors such as endothelial dysfunction (9), disorders of hemostasis (10), hemodynamic changes (11), and direct effects of thyroid hormones on the myocardium (12). Despite these associations, previous research on the link between thyroid dysfunction and CIMT has yielded inconsistent conclusions (13–16). There is a pressing need to explore the relationship between thyroid function and CIMT, enabling the early assessment of atherosclerosis and CVD.

Mendelian randomization (MR) is an innovative approach employing genetic variations, typically single-nucleotide polymorphisms (SNPs), associated with modifiable exposures (or risk factors) to assess their potential causal relationship with clinically relevant outcomes. MR aims to mitigate biases arising from confounding and reverse causation, especially in situations where randomized controlled trials are impractical and observational studies may introduce biases (17–19). Over the past decades, MR has emerged as a time-efficient and cost-effective tool for prioritizing potential factors influencing human biology and disease. In this study, we investigate the existence of a causal relationship between thyroid function and CIMT. Additionally, we employ multivariate MR to analyze potential mediating mechanisms.

## 2 Methods

### 2.1 Study design

**Figure 1:**
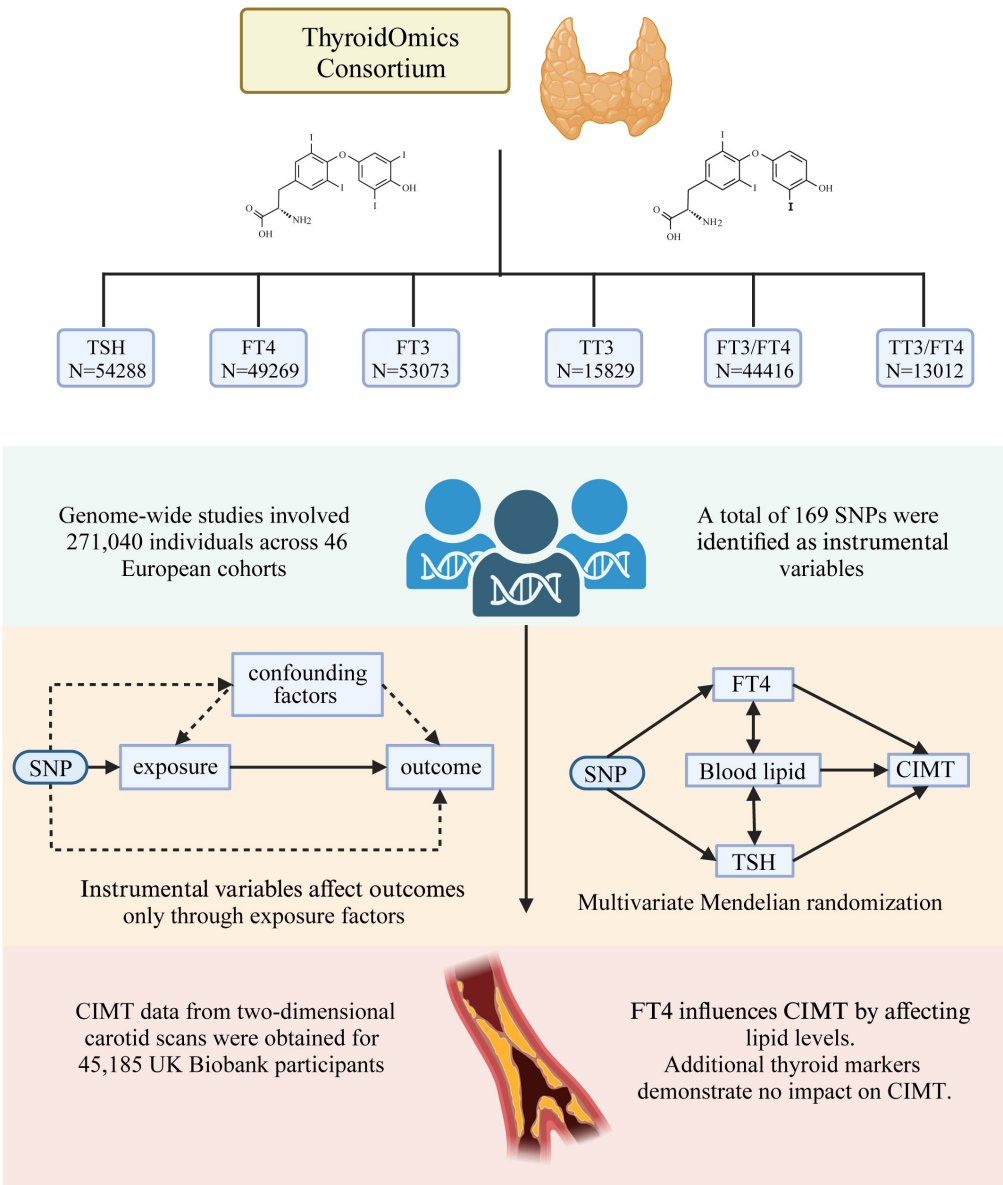
Overview of Study Design and Methodology. This figure illustrates the two-sample univariate Mendelian Randomization (MR) design utilized in our study to investigate the causal relationships between various thyroid function indicators and Carotid Intima-Media Thickness (CIMT). The analysis encompassed a wide range of thyroid function indicators, including thyrotropin (TSH), free tetraiodothyronine (FT4), free triiodothyronine (FT3), total triiodothyronine (TT3), FT3/FT4 ratio, TT3/FT4 ratio, and thyroid peroxidase antibodies (TPOAB), as well as conditions like hyperthyroidism and hypothyroidism. The study also evaluated the effects of levothyroxine treatment in hypothyroid patients and incorporated a multivariate MR analysis to assess the role of serum lipid profiles (apoB, apoA-I, HDL-C, LDL-C, and triglycerides) as potential mediators in the thyroid function-CIMT relationship. This figure was created by Biorender (https://www.biorender.com/).

The study employed a two-sample univariate MR design, as depicted in **Figure 1**, to elucidate the causal relationships between a comprehensive array of thyroid function indicators and CIMT. These indicators included thyrotropin (thyroid-stimulating hormone [TSH]), thyroxine (free tetraiodothyronine [FT4]), free triiodothyronine (FT3), total triiodothyronine (TT3) concentrations, FT3/FT4, TT3/FT4, thyroid peroxidase antibodies (TPOAB), hyperthyroidism, hypothyroidism. The research framework aimed to provide a detailed examination of the impact of these thyroid function indicators on CIMT, which included assessing the effects of levothyroxine treatment in hypothyroid patients. Recognizing the established association between lipids, thyroid function, and CIMT, the study also incorporated a multivariate MR analysis to evaluate the influence of serum lipid profiles, such as apolipoprotein B (apoB), apolipoprotein A-I (apoA-I), high-density lipoprotein cholesterol (HDL-C), low-density lipoprotein cholesterol (LDL-C), and triglycerides (TG), thereby exploring their potential role as mediators in this relationship.

Adhering to the three core assumptions of MR studies, the research ensured that the selected instrumental variables (SNPs) were associated with the corresponding phenotype, not linked to confounding factors affecting exposure and outcomes, and influenced outcomes exclusively through their impact on exposure. A meticulous screening process was implemented to select SNPs, with a particular focus on eliminating those with potential pleiotropic effects. Multiple sensitivity analyses were conducted to affirm the robustness of the study’s conclusions. The utilization of published abstract data from human participant studies negated the requirement for additional ethical approval and patient consent, streamlining the research process.

### 2.2 Source of data

The study integrated genome-wide association study (GWAS) datasets for TSH, FT4, T3, and the T3/FT4 ratio, obtained from 46 independent cohorts within the Thyroidomics Consortium. Participants under the age of 18, of non-European ancestry, those on thyroid medication, or with a history of thyroid surgery were excluded from all analyses. We collected information on the gender distribution, average age, and thyroid hormone parameter measurements across all cohorts (refer to **Figure 1**; **Supplementary Table 1**) (20, 21). SNPs representing TPOAB were derived from a meta-analysis of TPOAb GWAS involving 18,297 individual (22). Data on patients with hyperthyroidism (3,557 cases/456,942 controls) and hypothyroidism (30,155 cases/379,986 controls) were obtained from a meta-analysis of the UK Biobank and FinnGen (23). Data representing levothyroxine sodium users were sourced from the UK Biobank (18,947 cases/443,986 controls, id: ukb-b-17918). CIMT measurements were derived from two-dimensional carotid scans of 45,185 UK Biobank participants, encompassing a diverse ethnic background (24). Lipid-related traits, including LDL cholesterol, HDL cholesterol, triglycerides, apolipoprotein B, and apolipoprotein A-I, were sourced from a GWAS of circulating lipoprotein lipids by the UK Biobank (25).

### 2.3 Selection of genetic instruments

The process of selecting genetic instruments encompassed several steps: (1) screening for SNPs associated with exposure factors at genome-wide significance levels (*P* < 5 × 10^−8^) for TSH, FT4, hyperthyroidism, hypothyroidism, and thyroid hormone users, and a threshold of *P* < 5 × 10^−6^ for other thyroid function indicators to increase the availability of instrumental variables. For TPOAb, significant loci reported in the original literature were used; (2) Exclusion of SNPs exhibiting linkage disequilibrium (r2 = 0.001, kb = 10,000, 1000G EUR population); (3) alignment of the respective exposure and outcome datasets based on effect allele frequencies, excluding SNPs for intermediate alleles (EAF > 0.42); (4) assessment of instrument strength to mitigate bias from weak instrumental variables, evaluated using *F*-statistics. *F*-statistics for all extracted SNPs were above 10, signifying the absence of weak instrumental bias. SNPs with potential pleiotropic effects were identified and excluded using phenoscanner (http://www.phenoscanner.medschl.cam.ac.uk/) with a threshold of *P* < 5 × 10^-6^ (refer to **Supplementary Material** for details).

### 2.4 Mendelian randomization analysis

Univariate MR analyses were conducted to examine the causal effect of each exposure factor on outcomes. The primary analysis utilized multiplicative random effects inverse variance weighting (IVW-MRE). Additional MR methods, including MR-Egger regression and weighted median, were employed to corroborate IVW estimates and ensure robustness in various scenarios. MR-Egger regression, useful for detecting directional pleiotropy, and the weighted median method, offering consistent estimates in the presence of some invalid instrumental variables (26), were utilized. Multivariate MR analyses, incorporating lipid profiles, were performed to assess whether lipid factors mediated the effect of thyroid function on CIMT. To account for the linear relationship between LDL and apoB, HDL and apoA-I, the relationship between lipid profile, FT4, and TSH was corrected using the MVMR-Lasso method (27).

### 2.5 Sample independence

Sample independence is crucial to avoid weak instrument bias in MR analyses (28). We ensured no overlap between subjects in samples estimating genetic associations between exposure and results. Although some sample overlap existed between thyroid disease and carotid intima thickness, the strong statistical strength of subsequent MR studies (*F* > 10, see **Supplementary Information**) mitigated concerns of weak instrument bias.

### 2.6 Sensitivity analysis

MR analyses are potentially vulnerable to the influences of pleiotropy and heterogeneity, which can significantly skew results and lead to erroneous interpretations. In this study, we employed MR-Egger regression to detect and adjust for pleiotropy, a method that identifies and corrects for bias caused by gene variants affecting the outcome through pathways other than the exposure of interest (29). This approach is crucial for ensuring the validity of MR findings, as it addresses the concern that genetic variants may have multiple effects that confound the estimated exposure-outcome relationship. Additionally, Cochrane’s Q-test (30) was utilized to estimate and quantify heterogeneity among the genetic instruments. Assessing heterogeneity is essential to evaluate the consistency of the instrumental variable effects and to ensure the robustness and reliability of our findings. By addressing these potential sources of bias, our study enhances the credibility of the MR approach in elucidating causal relationships.

### 2.7 Statistical methods

In the primary analysis, Bonferroni correction was applied to account for multiple testing in two distinct parts of the study. For the analysis examining the relationship between thyroid function and CIMT, a Bonferroni-corrected threshold of P < 0.05/9 was employed. Similarly, for the analysis exploring the association between thyroid function and lipid profiles, a corrected threshold of P < 0.05/35 was used. Results achieving these Bonferroni-corrected thresholds were deemed significant, while correlations with P values between the corrected threshold and 0.05 were considered suggestive. MR results were expressed as effects (β) with corresponding 95% confidence intervals (CI), indicating the impact of each standard deviation increase in exposure factors on outcomes. Statistical analyses employed the TwoSampleMR software package (version 0.5.7) in R (version 4.3.1). Reporting adhered to STROBE-MR guidelines (18). For detailed information on instrumental variable screening and MR methods, refer to the **Supplementary Materials**.

## 3 Results

### 3.1 Instrument variable selection results

Through rigorous instrumental variable screening procedures, 10 SNPs were identified as instrumental variables for FT4, 33 SNPs for TSH, 2 SNPs for TPOAb concentration, 7 SNPs for hyperthyroidism, and 50 SNPs for hypothyroidism. Additionally, 27 SNPs were identified for FT3, 11 SNPs for TT3, 37 SNPs for the FT3/FT4 ratio, and 24 SNPs for the TT3/FT4 ratio (**Supplementary Table 2**).

### 3.2 Thyroid function and CIMT

**Figure 2:**
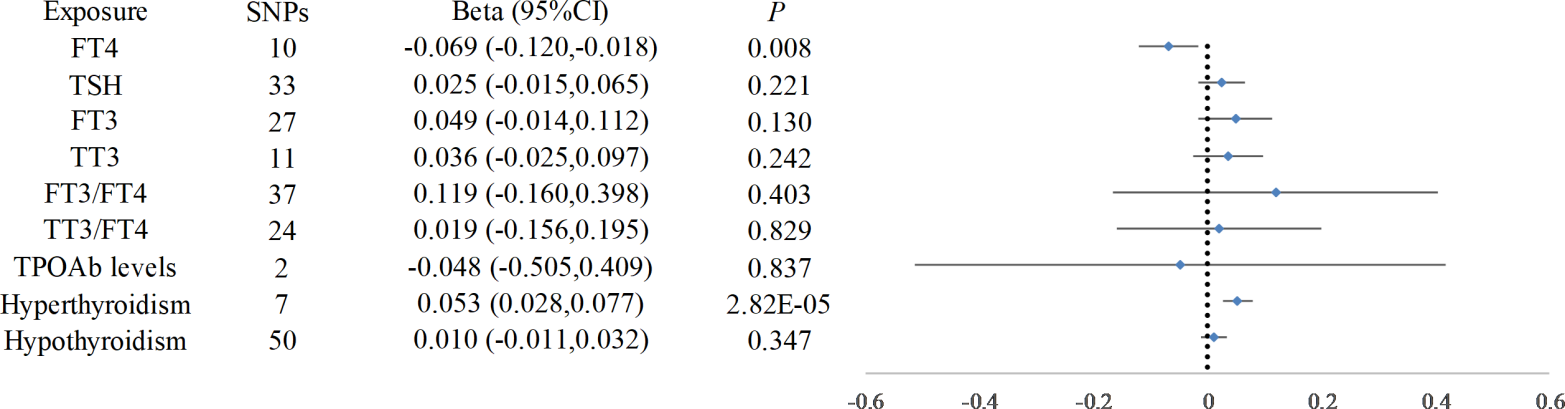
Causal Relationship Between Thyroid Function and Carotid Intima-Media Thickness. This figure presents the results of the Inverse Variance Weighted Multiplicative Random Effects (IVW-MRE) analysis, demonstrating a significant association between elevated FT4 levels and reduced CIMT. In contrast, FT3, TSH, and TPOAb concentrations showed no significant effect on CIMT. The figure also highlights the observed correlation between hyperthyroidism and increased CIMT, while hypothyroidism did not show a significant effect.

The IVW-MRE analysis demonstrated a significant association between elevated FT4 levels and reduced CIMT (beta = -0.069, 95% CI: -0.120, -0.018, *P* = 0.008). Conversely, FT3(beta = 0.049, 95% CI: -0.014 to 0.112, *P* = 0.130), TSH (beta = 0.025, 95% CI: -0.015 to 0.065, *P* = 0.221), and TPOAb concentrations (beta = -0.048, 95% CI: -0.505 to 0.409, *P* = 0.837) exhibited no significant effect on CIMT. Other thyroid function indicators did not impact CIMT (**Figure 2**). Hyperthyroidism was correlated with an increase in CIMT (beta = 0.053, 95% CI: 0.028 to 0.077, *P* = 2.8 × 10^−5^), whereas hypothyroidism did not demonstrate a significant effect on CIMT (beta = 0.010, 95% CI: -0.011 to 0.032, *P* = 0.347). Utilizing multivariate MR methods that accounted for hypothyroidism and thyroxin treatment, it was observed that hypothyroidism contributed to an increase in CIMT (beta = 0.106, 95% CI: 0.014 to 0.197, *P* = 0.023), while thyroxin treatment was associated with a decrease in CIMT (beta = -2.189, 95% CI: -4.287 to -0.091, *P* = 0.041) (**Table 1**).

**Table 1:** Thyroid function status and its impact on CIMT.

Thyroid Status	FT4 Levels	Impact on CIMT	Notes
Hypothyroidism	Low	No significant change	Thyroid hormone supplementation reduces CIMT thickness.
Normal Range	Normal	Decreased CIMT	Lower levels of FT4 within the normal range are associated with increased CIMT.
Hyperthyroidism	High	Increased CIMT	Hyperthyroidism is significantly associated with an increase in CIMT.

### 3.3 Thyroid function and lipid traits

**Figure 3:**
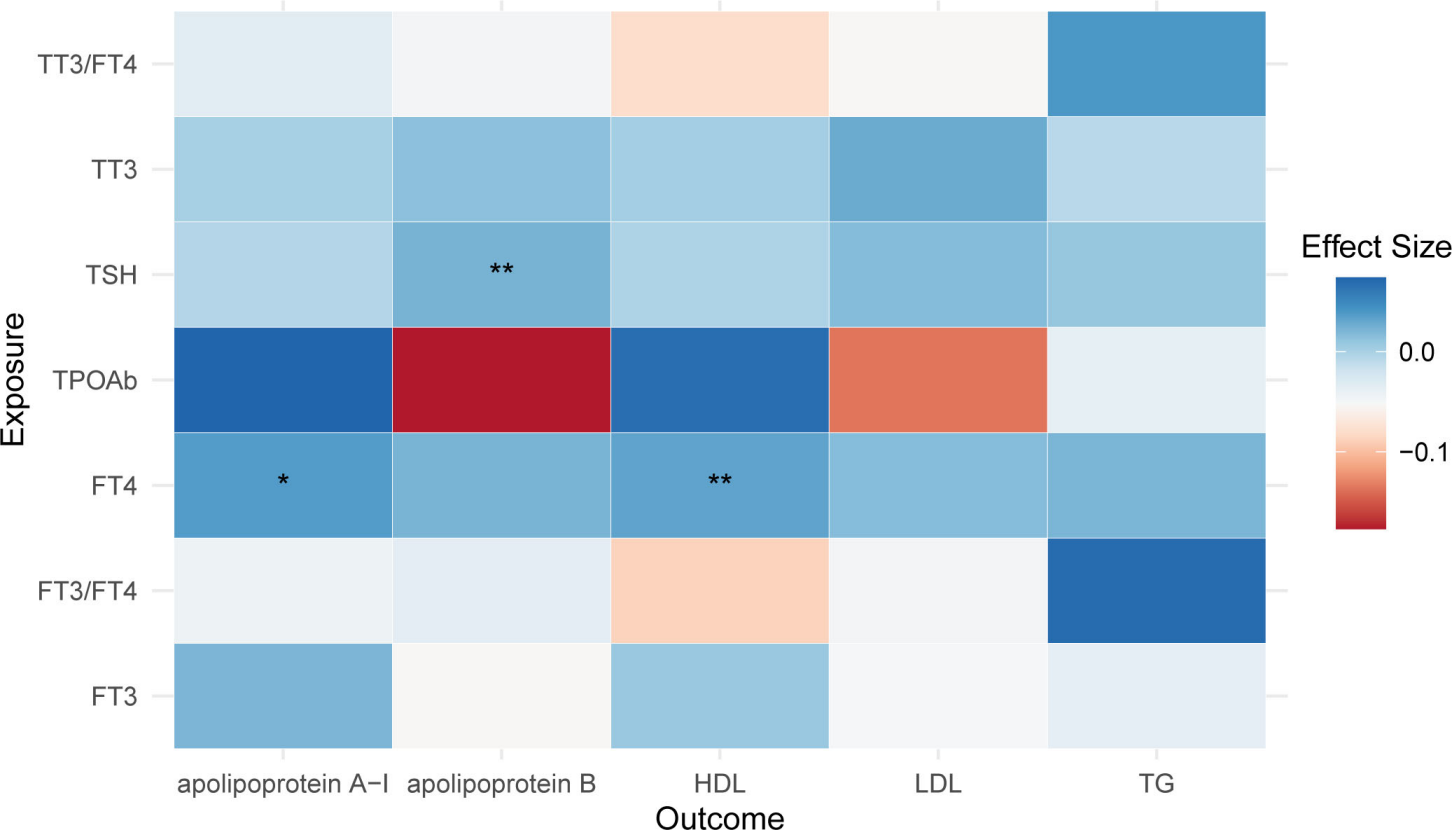
Causal Influence of Thyroid Function on Lipid Profiles. This figure demonstrates the effects of thyroid function on lipid profiles. An increase in FT4 is associated with higher levels of apoA-I and HDL, while a rise in TSH correlates with increased apoB levels. Notably, in this figure, asterisks indicate the level of statistical significance: a single asterisk (*) denotes a *P*-value less than 0.05, and double asterisks (**) denote a *P*-value less than 0.01.

An elevation in FT4 was observed to increase levels of apoA-I and HDL (apoA-I: beta = 0.037, 95% CI: 0.003, 0.072, *P* = 0.035; HDL: beta = 0.032, 95% CI: 0.009, 0.055, *P* = 0.006). Additionally, an increase in TSH was associated with a rise in apoB levels (beta = 0.022, 95% CI: 0.006, 0.038, *P* = 0.007). However, these findings did not meet the Bonferroni-corrected threshold. No impact of other thyroid function traits on lipid profiles was observed (**Figure 3**).

### 3.4 Results of multivariate MR

**Figure 4:**
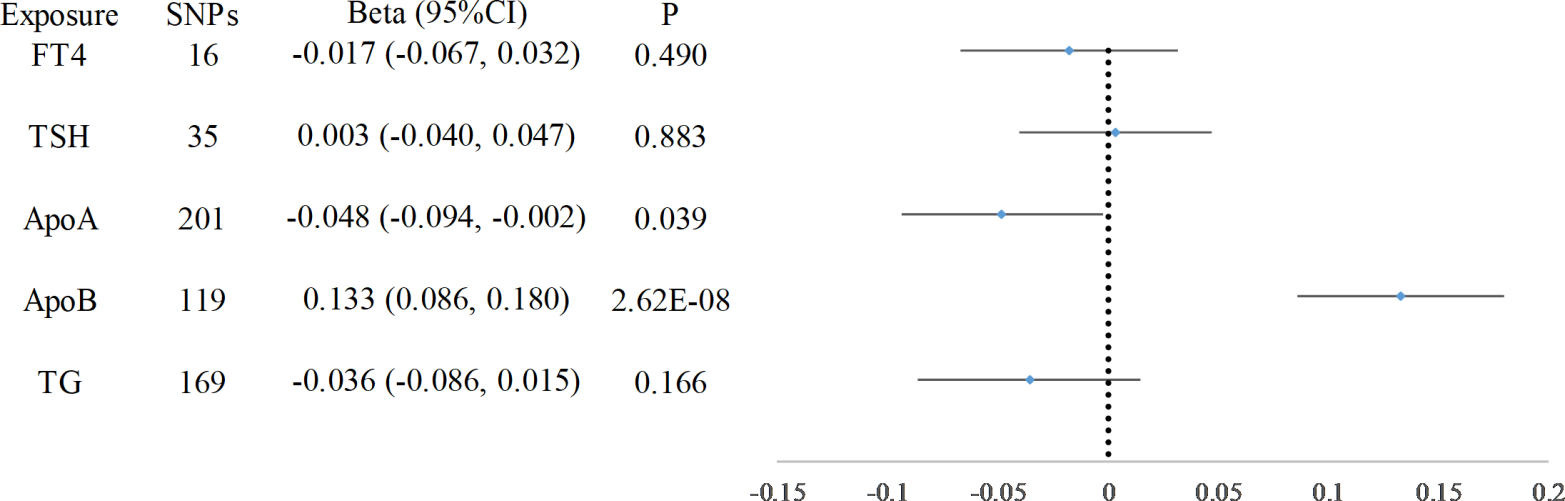
Multivariate MR Analysis Incorporating Lipid Factors. Image depicts the results of the multivariate MR analysis after adjusting for lipid traits, FT4, and TSH. The analysis revealed that only apoA-I and apoB were causally associated with CIMT, indicating their significant mediating role in the relationship between thyroid function and CIMT.

After adjusting for lipid traits, FT4, and TSH using multivariate MR, FT4 was no longer associated with CIMT. Only apoA-I and apoB were causally associated with CIMT (apoA-I: beta = -0.048, 95% CI: -0.094, -0.002, *P* = 0.039, apoB: beta = 0.133, 95% CI: 0.086, 0.180, *P* = 2.62× 10^−8^) (**Figure 4**).

### 3.5 Sensitivity analysis

Cochran’s Q test revealed heterogeneity in a subset of the results, particularly in the associations between TSH, FT3/FT4, and TT3/FT4 with CIMT. To mitigate the impact of this heterogeneity on the study outcomes, the IVW-MRE was employed. Additionally, the MR-Egger test, utilized for detecting pleiotropy, demonstrated intercepts near zero, suggesting an absence of pleiotropy in these associations (refer to **Supplementary Material** for details).

## 4 Discussion

This study represents the first application of MR methods to decipher the causal relationship between thyroid function and CIMT. Our two-sample MR analysis delved into the impact of thyroid disorders on CIMT. We discovered that lower FT4 levels within the normal range correlate with an elevation in CIMT. Conversely, FT3, TSH, and TPOAb exhibited no significant influence on CIMT. The administration of thyroid hormone supplements in hypothyroid patients emerged as a beneficial strategy to diminish CIMT, in contrast to the CIMT augmentation observed in hyperthyroidism. Further, our exploration into the interplay between thyroid function and lipid traits revealed a positive association of FT4 with apoA-I and HDL-C, and of TSH with apoB. Intriguingly, only apoA-I and apoB demonstrated a causal relationship with CIMT. This finding suggests a potential U-shaped curve relationship between FT4 levels and CIMT, mediated by apoA-I, underscoring the potential benefits of thyroid hormone supplementation in hypothyroid patients. Our research sheds light on the need to consider the risk of carotid atherosclerosis in the early routine evaluation of individuals with thyroid dysfunction.

Thyroid dysfunction, whether overt or subclinical, has been associated with increased CIMT (31); however, the precise mechanisms underlying this association remain elusive. It is plausible that thyroid-related biomarkers such as TSH, FT3, FT4, or TPOAb may contribute to endothelial dysfunction (32, 33). Moreover, thyroid dysfunction is often accompanied by metabolic disturbances, such as hyperlipidemia, obesity, and insulin resistance (34). These factors are established as clear risk factors for carotid atherosclerosis, leading clinical studies to primarily focus on patients’ metabolic abnormalities rather than changes in thyroid hormone levels.

Our findings highlight FT4 as a pivotal independent risk factor for increased CIMT. In cases of hypothyroidism, the administration of levothyroxine, a common thyroid hormone replacement therapy, has been shown to effectively reduce CIMT. We also observed that decreased FT4 within the normal range is associated with increased CIMT, aligning with previous clinical research (35, 36). Alessandro P. Delitala et al. found that higher FT4 levels increase arterial stiffness in the common carotid artery, possibly related to its effect on heart rate (37). In our study, we also noted increased CIMT in hyperthyroid patients. In addition, we discovered that the relationship between FT4 and CIMT is mediated by apoA-I, potentially elucidating the mechanism through which FT4 influences CIMT. However, Ayse S Cikim et al. found no significant difference in CIMT between subclinical hyperthyroid patients and the general population (38). Our study proposes a U-shaped relationship between FT4 levels and CIMT, underscoring the importance of routinely assessing thyroid function in patients to monitor the risk of carotid atherosclerosis. While our findings suggest a link between lower FT4 levels within the normal range and increased CIMT, current guidelines for subclinical hypothyroidism caution against the use of thyroxine therapy in individuals with TSH levels below 10 mIU/ml (39).

Previous studies have showed that FT3 is negatively correlated with coronary artery disease (40, 41), and higher FT3 is cross-sectionally associated with higher total HDL particle concentration and with lower HDL particle size in euthyroid individuals (42). Furthermore, T3 has been found to influence atherosclerosis through thyroid hormone receptors, which regulate the atherosclerotic process independently of the lipid profile (43). These findings underscore the importance of thyroid function in the context of atherosclerotic cardiovascular disease. Although our study found that FT3 has no significant effect on CIMT, a finding that may have resulted from data source limitations, the significance of FT3 for carotid atherosclerosis risk is still something that warrants careful consideration.

Serum TSH levels, sensitive to subtle changes in thyroid function, may promote atherosclerosis by stimulating the proliferation of vascular smooth muscle cells (39). Clinical studies have reported an association between TSH levels and CIMT in postmenopausal women (33), but no such association was found before menopause. Our study found no correlation between TSH levels and CIMT, however, an elevation in TSH was found to be positively associated with increased apoB levels. Therefore, it is important to acknowledge the potential impact of TSH on CIMT within specific subgroups.

TPOAb was associated with autoimmune diseases, and TPOAb titers correlate positively with high-sensitivity C-reactive protein levels (44). Prolonged inflammation may damage the process of endothelial repair, ultimately leading to atherosclerotic lesions. Shimizu, Y. et al. conducted a prospective study on 1069 Japanese subjects with thyroid hormones within the normal range, finding that an increase in TPOAb titers leads to progressive CIMT increase (45). However, TPOAb elevation often accompanies the onset of autoimmune diseases, making it challenging to analyze its direct effect on CIMT clinically. Our study did not find a correlation between TPOAb and CIMT, but caution is needed in interpreting this result, possibly due to the limited statistical power resulting from the inclusion of a small number of SNPs in the instrumental variable.

Apart from the influence of age and gender, CIMT is determined by traditional risk factors such as blood pressure and lipid abnormalities. There is an interaction between thyroid function and lipid metabolism, with MR studies revealing a causal relationship between changes in normal-range thyroid function and the diagnosis of metabolic syndrome and lipid status (46). The apoB/apoA-I ratio in hypothyroid patients is significantly higher than in those with normal thyroid function (47, 48). Clinical studies have shown that thyroid hormone supplementation can reduce lipids in patients with subclinical hypothyroidism (49), consequently lowering CIMT (35, 50).

To clarify the impact of the interaction between thyroid function and lipids on CIMT, we conducted a multivariable MR analysis. The results indicate that apoA-I, apo B, mediate the effect of FT4 on CIMT, emphasizing the crucial role of lipids in the process of CIMT thickening. Lipid-lowering drugs, as a frontline therapy for clinical prevention of carotid plaque formation and progression, are equally applicable to hypothyroid patients with increased CIMT. Our study also suggests that combined supplementation of thyroid hormones and lipid-lowering treatment may achieve a better reduction in CIMT for patients with hypothyroidism.

The primary strength of this study lies in its utilization of a MR design, where SNP assignment is randomized and unaffected by reverse causality, thereby minimizing potential bias from confounding factors. Moreover, the extensive sample size derived from GWAS summary statistics surpasses that of a typical RCT, enhancing the reliability and, to some extent, the predictiveness of the study’s outcomes regarding a clinical trial. However, several limitations should be acknowledged. Firstly, while MR provides insights into the long-term effects of thyroid hormones on CIMT, the results may not fully align with the outcomes of short-term drug interventions. The dynamics of physiological responses to exogenous hormones might differ from the prolonged impact of endogenous hormone levels. Secondly, limitations arise from the availability of instrumental variables. The scarcity of suitable SNPs for TPOAb may compromise the statistical power of the analysis. This constraint emphasizes the need for future research to identify additional instrumental variables for a more comprehensive understanding. Thirdly, the absence of individual-level data hindered a detailed exploration of the potential nonlinear relationship between FT4 and CIMT. Understanding such nuances could aid in establishing optimal hormone supplementation regimens. Fourthly, the study did not delve into the underlying pathophysiological mechanisms linking FT4 deficiency to CIMT. A deeper exploration of these mechanisms could enhance our understanding of the causal pathways involved. Finally, the generalizability of our findings is constrained by the study’s exclusive focus on populations of European ancestry. The applicability of our results to other ethnic groups remains uncertain and warrants consideration in future studies.

In conclusion, our findings suggest that lower FT4 levels may contribute to increased CIMT through their impact on lipid levels. While this study provides insights into the potential association between thyroid function and cardiovascular health, further investigations are warranted to ascertain the effectiveness of thyroid hormone supplementation in mitigating cardiovascular disease risk, particularly among individuals with subclinical hypothyroidism. These future studies should encompass diverse populations to ensure the broader applicability of the findings.

## Data availability statement

The original contributions presented in the study are included in the article/**Supplementary Material**. Further inquiries can be directed to the corresponding author.

## Author contributions

MZ: Conceptualization, Data curation, Writing – original draft. CZ: Data curation, Investigation, Software, Writing – original draft. XX: Data curation, Formal analysis, Writing – original draft. JL: Conceptualization, Project administration, Writing – review & editing.
